# Skin sensitization: Uncertainties, challenges, and opportunities for improved risk assessment

**DOI:** 10.1111/cod.13167

**Published:** 2018-12-07

**Authors:** Nicola Gilmour, Ian Kimber, Jason Williams, Gavin Maxwell

**Affiliations:** ^1^ Unilever Safety and Environmental Assurance Centre Bedford UK; ^2^ Faculty of Biology, Medicine and Health University of Manchester Manchester UK; ^3^ Contact Dermatitis Investigation Unit, Salford NHS Foundation Trust Salford UK

**Keywords:** differential susceptibility, health risks, induction and regulation, skin sensitization

## Abstract

At the ESCD congress held in Manchester in 2016, a session was organized to encourage more dialogue between clinicians with expertise in skin sensitization and toxicologists seeking to provide effective risk assessment to prevent human health issues. That session focused on the remaining uncertainties regarding the induction and regulation of skin sensitization in humans, and the opportunities and challenges associated with the refinement and improvement of risk assessment methodologies. This short article, prompted by those discussions, debates what the authors regard as being among the most important and most intriguing uncertainties about skin sensitization and allergic contact dermatitis in humans, and the most significant opportunities for improving risk assessment. The aim has been to provide a basis for mapping out the areas that might benefit from a closer alignment between the relevant clinical community and toxicologists charged with the responsibility of ensuring that skin sensitization risks are understood and managed.

## 1 BACKGROUND

Skin sensitization, resulting in allergic contact dermatitis (ACD), is a common occupational and environmental health problem. Skin sensitization is a T cell‐mediated hypersensitivity response that occurs if a susceptible individual is exposed to a quantity of a contact allergen sufficient to induce the activation, proliferation and clonal expansion of allergen‐responsive T cells. If the immune response is sufficiently vigorous, sensitization is achieved. Subsequent exposure of that now sensitized individual, at the same or a different skin site, to the same contact allergen can provoke an accelerated and more robust secondary immune response resulting in an inflammatory reaction at the site of contact that is recognized clinically as ACD. More detailed descriptions of this process are available elsewhere.^1^, ^2^, ^3^

What is known of the mechanism of acquisition of skin sensitization has been summarized in an adverse outcome pathway (AOP), which describes the main events (termed key events) leading from initial contact with a chemical (or other xenobiotic) to the induction of an adverse health effect.^4^, ^5^, ^6^ Despite this, there is much that is not known, or at least poorly understood, regarding the development of skin sensitization.

Many hundreds of chemicals have been shown to have the potential to induce skin sensitization, so there is a need to identify and characterize skin‐sensitizing chemicals and to accurately assess the health risks that may result from exposure. Human health risk assessments consist of four elements: (a) an assessment of consumer exposure, (b). hazard identification, (c) hazard characterization, and (d) risk characterization.^7^ The important exposure metric for the development of skin sensitization is the dose of chemical per unit area of skin,^8^, ^9^, ^10^ and this is the relevant exposure metric for risk assessment of contact allergens.

Hazard identification was originally conducted with guinea‐pig assays,^11^, ^12^ which were subsequently superseded by the mouse local lymph node assay (LLNA), a methodology that also permits hazard characterization for contact allergens.^13^ Recently, there has been substantial investment in the development, validation and application of novel non‐animal predictive test methods. This has been driven by ethical considerations, a changing regulatory landscape, and a desire to improve risk assessment. A number of assays have now been validated that are based on measurement of various key events leading to the development of skin sensitization,^14^, ^15^, ^16^, ^17^, ^18^, ^19^ and other methods are in the pipeline. A detailed description of the approaches is published elsewhere.^20^

For risk characterization, a point of departure (PoD) is first established. This is the point on the toxicological dose‐response curve corresponding to an estimated low effect level or no effect level. A safe reference dose is then derived that addresses sources of uncertainty that can arise in defining the PoD or in the extrapolation from experimental methods to real‐life exposures. The reference dose is then compared with the human exposure. In the area of risk assessment for skin sensitizers used in consumer products, the quantitative risk assessment (QRA) utilizes this process; the PoD corresponds to the no effect sensitization induction level, and the reference dose corresponds to the acceptable exposure level. Specific areas of uncertainty have been identified, and numerical safety assessment factors (SAFs) have been derived.^21^ These have recently been reviewed and revised, under the auspices of the International Dialogue for the Evaluation of Allergens (IDEA http://www.ideaproject.info) project,^22^ in order to be more explicit about what uncertainties are being accounted for within the risk assessment process. The key areas of uncertainty accommodated within QRA include interindividual variability, skin condition/body site, occlusion, vehicle or formulation effects, and frequency/duration of exposure. These have subsequently been subjected to review by the Scientific Committee for Consumer Safety, who note that some of these areas of uncertainty are underpinned by very limited data.^23^ Consequently, further research is needed if we are to address these data/knowledge gaps.

This is a time of transition in the toxicology of skin sensitization, as scientific advances, regulatory drivers and consumer preference all favour the replacement of traditional animal test methods with non‐animal alternative methods. The development of new approaches to hazard identification, and the need for new sources of information about potency and thresholds for the acquisition of skin sensitization, are driving new thinking about the process of risk assessment.

This short article aims to debate what the authors regard as being among the most important and most intriguing uncertainties about skin sensitization and ACD in humans, and to highlight the most significant opportunities for improving how uncertainty is addressed in the risk assessment process. It is our hope that this will provide a basis for mapping out the areas that might benefit from a closer alignment between the relevant clinical community and toxicologists charged with the responsibility of ensuring that skin sensitization risks are understood and managed.

## 2 SKIN SENSITIZATION: KEY UNCERTAINTIES, CHALLENGES, AND OPPORTUNITIES

### 2.1 How can non‐animal test methods best be deployed for the accurate identification of chemicals that have the potential to induce skin sensitization?

There are now available a number of validated *in vitro* methods that are recognized in Organization for Economic Cooperation and Development (OECD) test guidelines.^14^, ^15^, ^16^, ^17^, ^18^, ^19^ Moreover, a variety of other approaches, including those based on quantitative structure‐activity relationships, are in development or are currently undergoing validation (eg, References 24 and 25). Although these methods vary in their predictive accuracy, most provide a level of performance that is broadly comparable to that of *in vivo* tests, and it has been claimed some even show improved performance. One perceived limitation of the *in vitro* methods that have been validated to date is that they each seek to identify contact allergens as a function of a single key event in the AOP for skin sensitization. For this reason, the prevailing view currently is that these methods should not be used in isolation, but rather as part of integrated testing platforms that incorporate two or more separate assays, in, for example, defined approaches (DAs).^26^, ^27^, ^28^, ^29^, ^30^ Although this strategy might appear to be sensible, integration of different test systems has to be considered carefully if overall accuracy is going to be enhanced rather than compromised.^31^, ^32^ The evaluation of the non‐animal methods and DAs seeks to compare performance against the LLNA and human data; further collation and publication of both *in vitro* and benchmark *in vivo* datasets (including human data) is required to facilitate this evaluation process. Ultimately, however, consensus is required on how to combine the existing OECD test method data and other test data/information to reliably identify human sensitization hazards for all mechanisms of reactive chemistry, including the established challenge of identification of prehaptens and prohaptens.

### 2.2 How can non‐animal tests be used to provide information on the skin‐sensitizing potency of contact allergens?

Historically, the LLNA has been used to provide an accurate assessment of the relative skin‐sensitizing potencies of contact allergens. This has been possible because the LLNA provides a dose‐response curve by virtue of testing a series of test concentrations and measuring the response at each dose.^13^ The OECD test methods were developed and validated to allow the distinction between sensitizers and non‐sensitizers, so the question here is whether non‐animal methods (OECD Test Guideline methods or others), when used either alone or combined, can provide an estimation of human skin‐sensitizing potency to enable the use of a PoD in the risk assessment. Progress has been reported,^24^, ^25^, ^27^, ^28^, ^29^, ^30^, ^33^ but it is not yet clear how best to deploy such methods for potency assessment, how well they will perform in practice, and whether additional information or test methods will be required to inform a potency assessment, for example, measurement of the T cell response.^34^, ^35^, ^36^ Continued evaluation of DAs and integrated approaches to testing and assessment (IATAs) against benchmark in vivo (LLNA and/or human) data, and case studies evaluating risk assessment outcomes for historical materials with animal data with respect to risk assessment based on non‐animal data and clinical experience, are required to build a consensus on how to apply non‐animal data within DAs and IATAs for risk assessment decision‐making.^27^, ^28^

### 2.3 Is it necessary to increase our understanding of inherent immunoregulatory mechanisms that may serve to limit the extent to which sensitization is achieved to improve risk assessment?

An appreciation of the nature of immunoregulatory effects on human immune responses to contact allergens, and the influence of these on the acquisition of sensitization, is in its infancy.^37^, ^38^ There is no doubt that T cells play the most important roles in the acquisition of skin sensitization and the induction of ACD. However, the relative contributions (both positive and negative) made by discrete functional subpopulations of CD4^+^ (Th1, Th2 and Th17 subsets) and CD8^+^ T cells under different circumstances are not yet completely understood. Moreover, other cells of haemopoietic origin (including mast cells, natural killer cells, and granulocytes) may also be important.^6^, ^37^, ^39^, ^40^, ^41^, ^42^ By building an understanding of the immunological events that impact on the potency of sensitization responses in humans, and how different aspects of exposure, such as frequency, duration, and site of exposure, influence the ensuing immune response and the clinical manifestations, it might prove possible to enhance how we address these uncertainties within the risk assessment process.

### 2.4 Can a better understanding of how and to what extent inflammatory and danger signals influence skin sensitization refine our ability to address uncertainty relating to skin condition in risk assessment?

It has been recognized for many years that most contact allergens also have some potential to cause skin irritation and inflammation,^43^ and that simultaneous exposure to a non‐sensitizing skin irritant augments the acquisition of skin sensitization.^44^ However, we know little of the underlying mechanisms of this relationship. The uncertainty relating to how inflammation influences acquisition of skin sensitization is addressed within the QRA by the introduction of a SAF for skin condition at a given body site; the numerical values applied are pragmatic, and represent relative risks of the relevant body sites. The danger signals that are relevant for skin sensitization are damage‐associated molecular patterns (DAMPs).^45^ One important role of DAMPS, and of certain cytokines, is to trigger the activation, maturation and mobilization of dendritic cells (DCs),^46^ which are necessary for the induction of skin sensitization.^47^ The considerable heterogeneity among cutaneous DCs^48^, ^49^, ^50^, ^51^, ^52^ and their maturational status will have a significant impact on the nature of immune responses to chemical allergens, including the quality, vigour, regulation and longevity of those responses. The limited existing evidence suggests that enhancing our understanding of the role of inflammation in determining the vigour of immune responses could help us to understand the variations in sensitization potency shown by contact allergens, and enable further refinement of skin sensitization risk assessment.

### 2.5 Is our understanding of interindividual variation in susceptibility to skin sensitization fully accommodated in the risk assessment process?

The literature underpinning our current understanding of interindividual differences in susceptibility to skin sensitization has been reviewed previously.^22^ In short, there is little evidence to suggest that age has any major impact on the acquisition of skin sensitization, other than at the extreme age ranges (first year of life, and those aged >80 years)^53^, ^54^, ^55^, ^56^; the evidence on whether race is a significant factor in susceptibility to skin sensitization is weak,^53^ and there is only limited evidence for sex‐related differences in the acquisition of skin sensitization.^53^, ^54^, ^57^ However, experimental evidence from human studies shows that there are clear quantitative variations in responsiveness to defined contact allergens (such as 2,4‐dinitrochlorobenzene) that are expressed as differences in the level of exposure required to induce clinically discernible skin sensitization.^8^, ^58^ Provision for such differences is already made within the QRA process by the introduction of a SAF for human variability to account for the variation between a test population and a general population.^21^, ^22^

Variables other than those mentioned above might impact on the acquisition of skin sensitization, for instance: the effectiveness of the epidermal barrier, and differences in antioxidant systems and xenobiotic metabolic capacity.^53^, ^54^, ^59^ Under some circumstances, local metabolism/inactivation may reduce the ability of chemicals to induce skin sensitization.^60^, ^61^ Currently, it is not clear what the contribution of biotransformation in the skin is to the overall sensitizing potential and potency of chemicals in real‐world exposure scenarios. Furthermore, conditions associated with skewing of immune function can influence the effectiveness of skin sensitization.^62^ Lifestyle choices may also have more subtle effects, although these will probably be associated with changes in immune function. Finally, there may be other, as yet unrecognized, factors that impact on interindividual differences in susceptibility. An enhanced understanding of the relevance of some of these other, as yet unquantified, uncertainties could provide reassurance that existing practices are sufficiently protective, or provide new insights to improve our ability to quantify human variability.

### 2.6 Can a better understanding of critical exposure factors relating to susceptibility to acquisition of skin sensitization enable refinement of the risk assessment process?

In most cases (and with the exception of circumstances in which the area of exposure is very small), the important exposure metric for the development of skin sensitization is the dose of chemical per unit area of skin.^8^, ^9^, ^10^ However, a number of uncertainties remain, such as the impact of the frequency and anatomical site of exposure (including the possible influence of site[s] of exposure on the migration of antigen‐bearing DCs to regional lymph nodes), vehicle effects, and possibly even the timing of exposure in the context of biological body clocks and diurnal rhythms. Some of these will be more influential than others. Although some of these uncertainties are currently addressed within the QRA by either application of a SAF or incorporation of an assessment of aggregated dermal exposures, a better understanding of how vehicle impacts on localization within the skin and a better understanding of the real‐life exposures and risk factors that lead to the induction of skin sensitization could help to refine how we address some of these uncertainties.

### 2.7 Would better use of clinical data improve risk assessment?

This is a question that pervades much of what has already been discussed in this article. It is the view of the authors that a closer alignment between clinical experience of ACD (together with a more detailed understanding of the important factors that influence the development of skin sensitization in humans) and the toxicology community charged with conducting risk assessment and ensuring the safety of new products would yield significant benefits. This was one of the important themes to emerge from a discussion held at the ESCD meeting in Manchester in 2016. The value of clinical patch test data in informing risk management is well recognized,^63^, ^64^ but, as was highlighted during the ESCD 2016 discussions, toxicology risk assessors have limited access to such clinical insights, and rely on published data. This incurs a delay in the availability of information, and also limits information to materials that are already routinely patch tested for. Enhanced collaborations between clinicians and toxicologists would facilitate the identification of new allergens for patch testing, based on, for example, increases or changes in exposure, or when novel non‐animal data have been used to inform a risk assessment. Although it is a significant challenge to identify the original product causing induction of skin sensitization, it would be beneficial to more closely align and benchmark risk assessment outcomes with clinical findings. A better understanding of sensitizing exposures could enable refinement of the risk assessment to address increased risk.

## 3 CONCLUSIONS

The development of new approaches to hazard identification, and the need for new sources of information about potency and thresholds for the acquisition of skin sensitization, are driving new thinking about the process of risk assessment. Prompted by discussion between clinicians and toxicologists at the ESCD held in Manchester in 2016, we have identified some key uncertainties that should be addressed to improve next‐generation skin allergy risk assessments. Within the QRA, some uncertainties, such as intraindividual variability, matrix, and frequency and anatomical site of exposure, are already accommodated by assigning SAFs based on limited data and expert judgement. Further research will allow assessment of uncertainty to be more evidence‐based. The most significant opportunities are as follows:

- Evaluation of the predictive capacity of non‐animal methods and DAs, to reliably identify human sensitization hazards for all mechanisms of reactive chemistry.
- Evaluation of risk assessment outcome and clinical experience, to build a consensus on how to apply non‐animal data within DAs and IATAs for risk assessment decision‐making.
- Building an understanding of how exposures such as frequency, duration and site of exposure influence the ensuing immune response and the clinical manifestations, to enhance how we address these uncertainties within the risk assessment process.
- Enhanced understanding of the role of inflammation in determining the vigour of immune responses, to enable further refinement of skin sensitization risk assessment.
- Enhanced understanding of other variables that might impact on the acquisition of skin sensitization, to provide reassurance that existing practices are sufficiently protective or provide new insights to improve our ability to quantify human variability.
- Enhanced understanding of how exposure might influence skin sensitization, for instance, how vehicle impacts on localization within the skin, and a better understanding of the real‐life exposures and risk factors that lead to the induction of skin sensitisation, to help refine how we address some of these uncertainties.
- Enhanced collaborations between clinicians and toxicologists, to facilitate the identification of new allergens for patch testing, based on, for example, increased or changed exposure or when novel non‐animal data have been used to inform a risk assessment.

Through a closer alignment between the relevant clinical and the toxicology communities, and further research and collaboration, we can endeavour to provide effective next‐generation risk assessment to prevent the development of human health issues. To achieve this aim, collaborative forums (either existing or new) need to evolve to bring the two communities together to address some of these questions.

## CONFLICTS OF INTEREST

The authors have no conflicts of interest to report. The authors alone are responsible for the content and writing of this article.
